# A novel human in vitro papillomavirus type 16 positive tonsil cancer cell line with high sensitivity to radiation and cisplatin

**DOI:** 10.1186/s12885-019-5469-8

**Published:** 2019-03-25

**Authors:** Ola Forslund, Natsuki Sugiyama, Chengjun Wu, Naveen Ravi, Yuesheng Jin, Sabine Swoboda, Fredrik Andersson, Davit Bzhalava, Emilie Hultin, Kajsa Paulsson, Joakim Dillner, Stefan Schwartz, Johan Wennerberg, Lars Ekblad

**Affiliations:** 1 Department of Laboratory Medicine, Division of Medical Microbiology, Skane Laboratory Medicine, Lund University, Lund, Sweden; 2 Department of Clinical Sciences Lund, Oncology and Pathology, Lund University, Skane University Hospital, Barngatan 4, SE-222 25 Lund, Sweden; 3 Department of Laboratory Medicine, Division of Clinical Genetics, Lund University, Lund, Sweden; 4 Department of Clinical Sciences Lund, Oto-rhino-laryngology, Head and Neck Surgery, Lund University, Skane University Hospital, Lund, Sweden; 5 Department of Pathology, Regional Laboratories Region Skane, Skane University Hospital, Lund, Sweden; 6 Department of Laboratory Medicine, Division of Pathology, Karolinska Institutet, Stockholm, Sweden

**Keywords:** Tonsil cancer, Human papilloma virus, Cell line, Cisplatin, Radiation, Cetuximab

## Abstract

**Background:**

Human papillomavirus (HPV) is an established risk factor for oropharyngeal squamous cell carcinoma (OSCC). The aim was to establish cell lines from HPV-positive tonsil carcinomas to be used for treatment development.

**Methods:**

Fresh samples from 23 HPV-positive tonsil carcinomas were cultivated in vitro. The established cell line was analyzed for viral characteristics, cell karyotype, *TP53* status, and growth capabilities in nude mice. In vitro studies of sensitivities to radiation, cisplatin and cetuximab were performed.

**Results:**

After 19 months (eight passages), one cell line, LU-HNSCC-26, was established in vitro and also grew as xenografts. The tumor was from a 48 year old non-smoking man with non-keratinizing, p16 positive tonsil OSCC, stage T2N0M0 with HPV16. It contained 19.5 (CV% 3.7) HPV16 copies/cell (passage 8). The complete HPV16 genome sequence was obtained. Episomal HPV16 was present with an E2/E7 ratio of 1.1 (CV% 2.6). In addition, HPV16 mRNA specific for the intact E2 gene was detected. The viral expression manifested 1.0 (CV% 0.1) E7 mRNA copies per HPV16 genome. The karyotype was determined and the cell line demonstrated wild type *TP53*. The ID50 for radiation was 0.90 Gy and the IC50 for cisplatin was 0.99 μmol/L. The cell line was inhibited to a maximum of 18% by cetuximab.

**Conclusions:**

We established an in vitro tonsil carcinoma cell line containing episomal HPV16. This is an important step towards efficient treatment development.

**Electronic supplementary material:**

The online version of this article (10.1186/s12885-019-5469-8) contains supplementary material, which is available to authorized users.

## Introduction

In recent years, there has been an increase in the incidence of oropharyngeal squamous cell carcinoma (OSCC) including cancers in the tonsils and the base of the tongue [1, 2]. In Sweden, the increase has been close to 5% per year between 1970 and 2016 [3] which represents a doubling in incidence every 14 years. The patients in this group are generally younger and often lack the traditional risk factors for head and neck squamous carcinoma (HNSCC) such as a history of smoking and alcohol abuse [4].

There is strong evidence that this rise in OSCC incidence is caused by human papillomavirus (HPV) infection [1] and that this also explains an observed shift to higher survival rates for this cancer type [5] as patients with HPV-positive OSCC have a better overall survival and better treatment response compared to patients with HPV-negative OSCC [6].

In many respects, including incidence, survival and treatment response, HPV-positive and HPV-negative OSCC behave differently, and it has become clear that the treatment strategies should differ significantly between the groups and that new regimes should be developed specifically for HPV-positive OSCC.

An important tool for testing new drugs or combinations of drugs as well as for the elucidation of the biology of HPV-induced transformation is representative, in vitro and in vivo, HPV-positive OSCC cell lines. So far, a small number of HPV16-positive HNSCC cell lines have been established in vitro but none originating from tonsil tumors and all from patients with a history of smoking and alcohol use [7, 8]. As the outcome is different for tumors linked to HPV compared to those linked to the classical risk factors, and for HPV-positive tumors in the oropharynx (especially in the tonsils) compared to those originating in other sites [9–11], it is very important to establish cell lines from tonsil tumors in order to perform valid pre-clinical therapy development.

For uterine cervical cancer, the integration process has been shown to be of great importance for the carcinogenic process, though not being an exclusive prerequisite as episomal HPV16 was present in 47% of cervical HPV16-positive cancers, either as pure episomal (27%) or as a mixture of integrated and episomal HPV16 (20%) [12]. Measurements of the physical state of the HPV genome in OSCC have shown very different frequencies of integration, possibly as a consequence of a combination of different methods used to assess integration status and varying sample sizes. But it has been reported that, among HPV16 positive cases, 76% of OSCCs and 93% of tonsil carcinomas manifest episomal HPV16 either as pure episomal or as the mixed status [13–15], thus implicating that the frequency of the episomal state of HPV16 is more common in OSCC than in cervical cancer. To the best of our knowledge, no cell line with episomal HPV16 derived from tonsil carcinoma has been established in vitro.

The aim of the presented work was to establish cell lines in vitro from HPV-positive OSCC tumors that could be used for therapy development and basic research purposes.

## Materials and methods

### Patients

The study was approved by Lund University regional ethical review board (LU 376–01), and the samples were collected, after informed consent was obtained, at Skåne University Hospital, Lund, Sweden.

### Establishment of cell line in vitro

Tumor samples were rinsed consecutively in phosphate buffered saline (PBS) and R10 medium (RPMI 1640 with stable glutamine supplemented with 1 mmol/L sodium pyruvate, 1 × MEM non-essential amino acids, 20 μg/mL gentamicin and 10% fetal bovine serum (FBS), all from GE Healthcare (Piscataway, NJ, USA). The tumor samples were cut in pieces and transferred to cell culture flasks with R10 medium and left to attach and grow at 37 °C under a humidified atmosphere with 5% CO_2_. The cells used in the described experiments were passaged less than 18 times after establishment. Mycoplasma tests were performed on a regular basis by GATC Biotech AG (Konstanz, Germany) and were negative.

For cell doubling determination, cells were seeded in eight 96-well plates. The plates were analyzed by the sulphorhodamine B (SRB) method as previously described [16] consecutively at the indicated time points. Error bars indicate standard error of the mean. The data points were fitted to a logarithmic equation using the GraphPad Prism (5.04) software package (GraphPad Software, La Jolla, CA, USA).

### Single tandem repeat analysis

Single tandem repeat (STR) analysis was performed by Uppsala Genome Center using the AmpFlSTR Identifiler polymerase chain reaction (PCR) amplification kit (Applied Biosystems, Carlsbad, CA, USA). Separation of DNA fragments was performed with capillary electrophoresis on the ABI3730XL DNA analyzer (Applied Biosystems, Foster City, CA, USA) and allele scoring was performed with the GeneMapper 4.0 software package.

### Establishment of xenograft

Experiments on mice were approved by the local ethics committee (488/2017). We used in-house bred, athymic 5- to 8-week-old BALB/c nude (nu/nu) mice. The mice were given food and water ad libitum and treated according to regulations issued by the Swedish Board of Agriculture (SJVFS 2017:40). Cells were injected subcutaneously into the flanks of non-anesthetized animals. To determine the doubling time, tumors were retransplanted to 10 nude mice under ketamine anesthesia, two tumors per mouse. The length and width of the tumors were measured with calipers three times per week and the relative tumor size calculated in relation to day 0 (set as the day when all tumors had shown growth over 3 consecutive time points). The data points were fitted to a logarithmic equation in GraphPad Prism. After the measuring period, the mice were euthanized using cervical dislocation and the tumors removed for histological sectioning.

### Histology

Xenografts and cell pellets were sectioned in 3 μm slices and stained with hematoxylin and eosin, and the following antibodies: the CINtec p16-kit (E6H4, cat. no. 725–4713), anti-pan keratin, (AE1/AE3/PCK26, cat. no. 760–2595), and anti-p63 (4A4, cat. no. 790–4509) all from Ventana Medical Systems (Tucson, AZ, USA), anti-cytokeratin 5 (XM26, cat. no. NCL-L-CK5) from Leica Biosystems (Nussloch, Germany), and p40 (BC28, cat. no. ACI3066C) from Biocare Medical (Pacheco, CA, USA), All stainings were performed using the BenchMark Ultra platform (Ventana Medical Systems).

### Cell lines

Three HNSCC cell lines, previously established in our laboratory, were used for comparison in several experiments: LU-HNSCC-4, LU-HNSCC-5 and LU-HNSCC-6 [17]. They were propagated in Dulbecco’s modified Eagle’s medium (DMEM) supplemented with 10% FBS, 100 units/mL penicillin, and 100 units/mL streptomycin sulfate, and used within 15 passages after thawing. Single tandem repeat analysis was performed before the experiments as detailed above showing no cross-contamination between the cell lines or with other common contaminants. Tests for mycoplasma infection prior to the experiments were negative.

### DNA extraction

A 2–3 mm biopsy was immersed in 1 mL saline solution and immediately transferred to the laboratory. The saline was removed and the biopsy was digested in lysis buffer and the DNA extracted with the Total NA-kit using MagNA Pure LC [18] (Roche, Pleasanton, CA, USA). Sample adequacy was assessed by PCR for the human beta-globin gene [19]. Identification of 39 genital HPV types was carried out by modified general primer polymerase chain reaction (MGP-PCR) and subsequent Luminex analysis (Luminex, Austin, TX, USA) [18, 20, 21].

Samples from subsequent passages in cell cultures were immersed in 1 mL GITS (4 mol/L guanidinium thiocyanate, 22 mmol/L sodium citrate, and 5% N-lauroylsarcosine sodium salt) and stored at − 20 °C. After thawing, 200 μL was used for DNA extraction with the Total NA-kit using MagNA Pure LC (100 μL output).

### Viral load

Briefly, the number of viral genomes per cell was quantified by carrying out two separate real-time PCR tests, including appropriate standard curves, to amplify the HPV16 E7 gene and the human beta-globin gene as previously described [18]. The samples were analyzed in duplicate.

### Physical status of HPV16

As a marker for the presence of mixed or episomal forms of HPV16, samples were analyzed for the ratio of E2/E7 gene copy numbers. HPV16 was classified as mixed status when complete E2 was evident from complete sequencing of the HPV16 genome and the E2/E7 ratio was < 0.9, and pure episomal status in the presence of complete E2 open reading frame (ORF) and E2/E7 ratio of > 0.9. The E7 copy numbers were retrieved from the viral load measurement as described above. For quantification of the E2 copy numbers we used a modified version of the PCR described by Peitsaro et al. [18], where E2/E6 ratios greater than 1 indicate predominance of the episomal form [22]. Quantification was extrapolated from a linear regression standard curve that was included in each batch assay, as previously described [18]. The samples were analyzed in duplicate.

### Long range HPV16 PCR

The complete HPV16 genome was amplified in a 25 μL solution with 2.5 μL (15.5 ng) of extracted nucleic acid from passage 2, 0.2 μmol/L of each primer (HPV16 7465F, 5′-ATGCTTTTTGGCACAAAATGTG, HPV16 7464R 5′-GCAACCGAATTCGGTTGAAG), 200 μmol/L of each dNTP, 1 × PrimeStar GXL Buffer, and 0.625 U PrimeStar GXL DNA Polymerase (TaKaRa Bio, Shiga, Japan). The PCR was performed at 98 °C for 10 s, 60 °C for 10 s, and 68 °C for 8 min for 45 cycles. Amplified DNA was separated by electrophoresis in a 1.0% agarose gel (agarose Type I-A, A0169, Sigma-Aldrich, Stockholm, Sweden) with 1 × Gel RedTM Nucleic Acid Gel Stain (VWR International, Lund, Sweden) in 0.5 × TBE buffer [23] and visualized by UV light.

### DNA sequence of the complete HPV16 genome

The complete HPV16 genome was obtained by next-generation sequencing (NGS) in the MiSeq system (Illumina, San Diego, CA, USA) from passage 2.

Briefly, extracted DNA (5.6 ng/μL in a total volume of 20 μL) was whole genome amplified (WGA) with multiple displacement amplification using the Illustra™ Ready-To-Go™ GenomiPhi™ DNA Amplification Kit (GE Healthcare) based on the manufacturer’s protocol, but with some modifications. Five microliter of extracted DNA was diluted with 20 μL PCR-grade water and 25 μL denaturation buffer, incubated at 95 °C for 3 min, cooled on ice, added to the lyophilized cake, incubated at 30 °C for 7 h and inactivated at 65 °C for 10 min. The WGA product was diluted 1:2 in PCR-grade water and quantified with QuantiFluor-ST (Promega, Madison, WI, USA). A DNA library with 2 unique indices was prepared from 50 ng of the WGA product using the Nextera DNA Sample Preparation kit, user guide revision B (Illumina) and validated by fragment size using 2100 Bioanalyzer High sensitivity DNA assay (Agilent Technologies, Santa Clara, CA, USA) and quantified with QuantiFluor-ST (Promega). The library was normalized to 4 nmol/L in EB-buffer (Qiagen, Hilden, Germany), denatured, diluted to 1.8 pmol/L, spiked with 1% PhiX control and paired-end sequenced by 151 plus 151 cycles using NextSeq 500 High Output reagent kit (Illumina) as described in the user guides *Denaturing and Diluting Libraries for the NextSeq 500*, revision A and *NextSeq 500 kit Reference Guide*, revision F.

Papillomavirus-related contigs having > 90% identity with each other were clustered. The total number of sequencing reads from each cluster was calculated and the longest contig was selected as a representative to remove redundancy. A de novo HPV-sequence was obtained by the use of clustering analysis, conducted separately for contigs longer and shorter than 700 bp length. For the HPV type, the total number of reads was calculated. All analyses were performed using in-house R (http://www.r-project.org/), python (http://www.python.org) and bash (http://www.gnu.org/software/bash/) scripts that ran on a high performance (40 core, 2 TB RAM) Linux server.

The obtained sequence was then analyzed by the use of BioEdit v7.0 [24] for presence of the expected ORFs and in cases of ambiguities, re-sequencing from the passage 2 was performed to resolve the ambiguity.

The primary de novo sequence had two segments of N-ambiguities of 72 bases and of 129 bases at positions 4633–4704 and 7150–7278, respectively. In order fill these gaps, two PCRs were performed, each with 50 μL PCR mixture that contained 5 μL of template of extracted DNA from passage 2, 0.3 μmol/L of each forward and reverse primer, 200 μmol/L of each dNTP (Roche), 1.5 U AmpliTaq Gold, 1 × reaction buffer and 3.0 mmol/L MgCl_2_ (all from Applied Biosystems). The following primer pairs were used to generate amplicons of 887 bp with HPV16 forward primer at position 3992 (5′- TATTGTGGATAACAGCAGCCTCTG) and reverse primer at position 4878 (5′- GGTGTGCTACTAGTTACTGTGTTAGGG), and of 316 bp with HPV16 forward primer at position 7048 (5′-AAGCAGGATTGAAGGCCAAAC) and reverse primer at position 7363R (5′- AATAACCACAACACAATTAGTAGGTGTTG). We also separately sequenced a fragment of 250 bp in order to verify the guanosine at position 1376, by a HPV16 forward primer at position 1250 (5′- GCGAAGACAGCGGGTATGG) and a reverse primer at position 1489 (5′- TGCTAACATTGCTGCCTTTGC).

PCR was carried out in an automated thermocycler (Eppendorf) programmed for tube setting: with the following parameters: 94 °C for 10 min; 45 × (95 °C for 15 s, 55 °C for 30 s, and 72 °C for 1 s). Five microlitres of the amplicons were separated by electrophoresis in a 2.0% agarose gel (agarose Type I-A, A0169, Sigma-Aldrich) with 1 × Gel RedTM Nucleic Acid Gel Stain (VWR International, Lund Sweden) in 1 × TBE buffer [23] and visualized by UV light. The remaining amplified material (45 μL) was purified by Illustra MicroSpin S-300 HR Columns (GE Healthcare) and sequenced with the HPV16 primers described above (Eurofins, Ebersberg, Germany). The sequences were then used to fil the gaps of the de novo sequence and the final sequence was compiled.

### mRNA extraction and quantitative mRNA RT-PCR

Cells were immersed in GITS and used for mRNA extraction with the Oligotex Direct mRNA Mini Kit (Qiagen) [18]. The quantitative mRNA PCR was performed as previously described [18].

### RNA extraction and RT-PCR

Total RNA was extracted using TRI Reagent (Sigma-Aldrich) and Direct-zol RNA MiniPrep (ZYMO Research, Irvine, CA, USA) according to the manufacturer’s protocol. One μg of total RNA was reverse transcribed in a 20 μL reaction at 37 °C by using M-MLV Reverse Transcriptase and random primers (both from Invitrogen, Carlsbad, CA, USA) according to the protocol of the manufacturer. One microliter of cDNA was subjected to PCR amplification with primers indicated in Fig. 3 and listed in Table 1.

**Table 1:** Reverse-transcription PCR primers for amplification of HPV16 mRNA

Oligo number	Oligo name	Oligo sequence
1	P97s	GTCGACCTGCAATGTTTCAGGACCC
2	773 s	GCACACACGTAGACATTCGTACTTTG
3	E4as	TGCTGCCTAATAGTTTCAGGAGAGG
4	E2as	CCTGACCACCCGCATGAACTTCC
5	Set3-F	GACCCCCTTTAACAGTAGATCC
6	Set3-R	TACAGATGGGTCAGTGAAAGTG
7	E4s	GCCCTCTCCTGAAACTATTAGGCAGCA
8	Llas	GCAACATATTCATCCGTGCTTACAACC

### Protein extraction and Western blotting

Cells were lysed in radioimmunoprecipitation assay buffer (50 mmol/L Tris, pH 7.8, 150 mmol/L NaCl, 1% sodium deoxycholate, 0.1% sodium dodecyl sulphate, 1% Triton X-100, 1 mmol/L dithiothreitol and protease inhibitors), subjected to SDS-PAGE, transferred to nitrocellulose membrane, and stained with antibodies against HPV16 L1 (ab30908, Abcam, Cambridge, UK), L2 (sc-65,708, Santa Cruz Biotechnology, Dallas, TX, USA), and epidermal growth factor receptor (EGFR) (no. 2232, Cell Signaling Technology, Danvers, MA, USA). Proteins were detected using chemiluminescence. For quantification of EGFR, loading control was performed using Coomassie R-350 as previously described [25].

### Screening of mutations in *TP53*

Primers for all exons of *TP53* were designed using the Primer3 software (http://primer3.ut.ee/); primer sequences are available on request. PCR was done on genomic DNA according to standard methods and amplified fragments were sequenced using the BigDye v1.1 Cycle Sequencing Kit on an ABI-3130 Genetic Analyzer (Applied Biosystems). ChromasLite 2.1.1 free online software (http://technelysium.com.au/) was used to analyze the data.

### Cytogenetic analysis

For cytogenetic analysis, cells were cultured in DMEM/F12 medium with 10% fetal bovine serum and antibiotics until the mitotic index was high, and then exposed to colchicine (0.03 μg/mL) for 4–5 h. The cells were detached by trypsin-EDTA, treated with 0.06 mol/L KCl for 35 min, and then fixed three times with methanol/acetic acid 3:1. Chromosome preparations were incubated at 60 °C overnight and then treated with 0.3 mol/L sodium chloride, 30 mmol/L sodium citrate, pH 7.0, at 60 °C for 4 h. G-banding was done using Wright’s stain and karyotypes were described according to ISCN 2009 [26].

### Determination of treatment sensitivity

For cisplatin, cells were seeded and, after 48 h, treated with increasing concentrations of cisplatin (Sandoz AS, Copenhagen, Denmark) for one hour. After 5 days at 37 °C under a humidified atmosphere with 5% CO_2_, the cell amounts were measured by the SRB assay.

For determination of cetuximab sensitivity, cells were treated with increasing concentrations of cetuximab (Merck AB, Solna, Sweden) for 5 days, after which the cell amounts were determined by the SRB assay.

Radiosensitivity was analyzed by irradiating the cells in a Gamma Cell-3000 Elan from MDS Nordion (Ottawa, ON, Canada) equipped with a Cs-137 source. After irradiation, the cells were seeded and left to grow until the untreated controls were approximately 90% confluent, and then analyzed by the SRB assay.

For all experiments the data were fitted to a sigmoidal dose-response equation with variable slope using non-linear regression, and the half maximal inhibitory concentration (IC_50_), inhibitory dose (ID_50_) or maximum inhibition (E_max_) calculated in GraphPad Prism.

## Results

### Establishment of cell line

A total of 27 tumor samples (23 HPV-positive), were collected and treated as detailed in the Materials and Methods section. In a single case, we were able to establish a cell line. The original tumor tissue was obtained from a 48 year old non-smoking man with moderate daily consumption of alcohol, and, besides exercise induced asthma, no other intercurrent diseases. The initial symptom was left-sided pain in the throat. He had no weight loss. Three days after his first symptom he was examined by an ENT-specialist who noted a prominent left sided tonsil without mucosal ulceration. No nodes could be felt. A fine-needle aspirate was performed, with negative result. Two and a half weeks later he was examined under anesthesia and biopsied through intact mucosa. This biopsy confirmed a non-keratinizing, p16 (CDKN2A) positive squamous cell tonsil carcinoma with HPV16 detected in the original biopsy. After further investigation with CT and CT-PET scan, the tumor was classified as stage T2N0M0. The patient was treated with full dose radiotherapy (Intensity-modulated radiation therapy) without concomitant chemotherapy leading to full remission, and was relapse-free after four and a half years. The primary tumor received 64 Gy (2.0 Gy/fraction) and the neck 54.4 Gy (1.6 Gy/fraction).

**Fig. 1:**
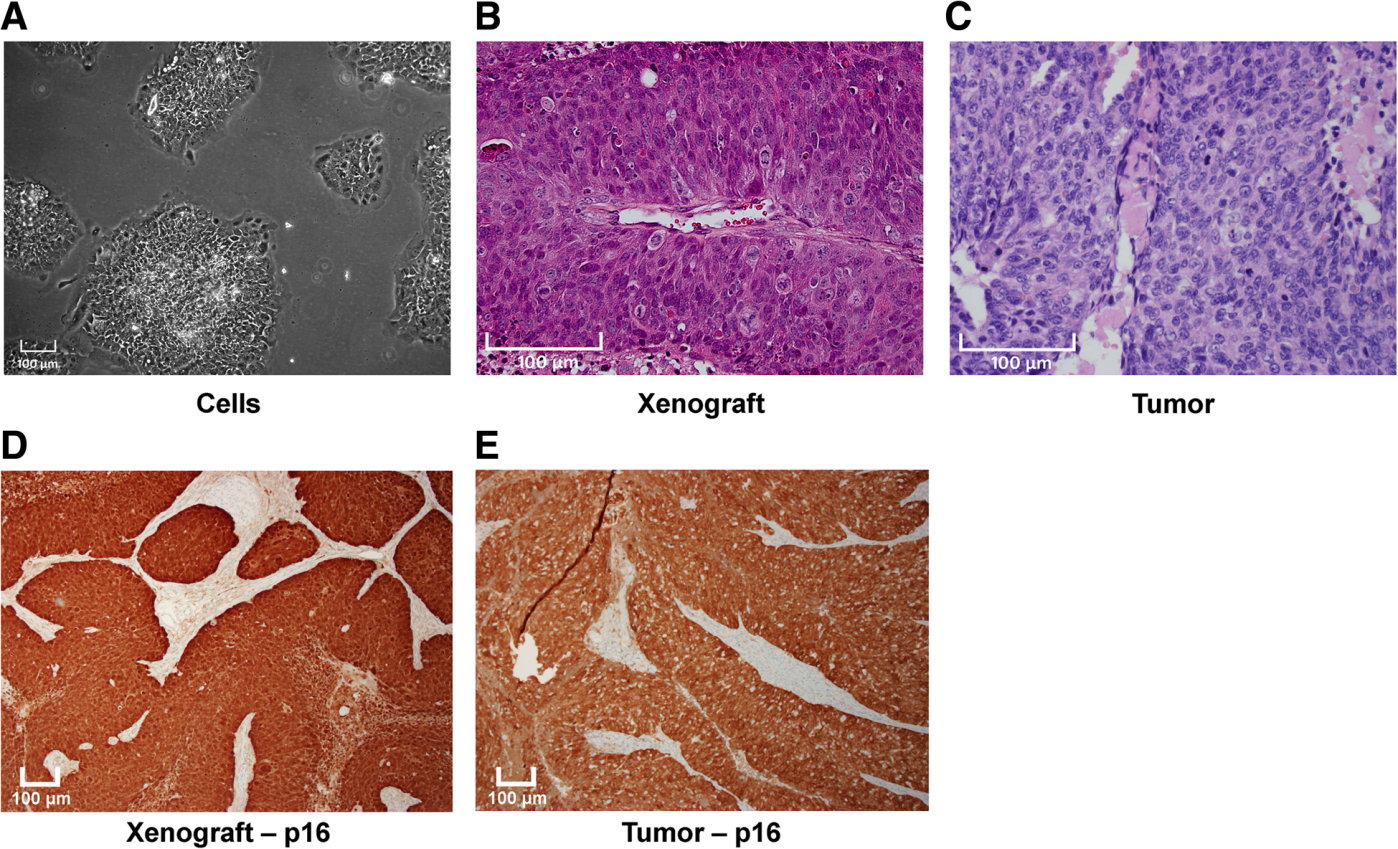
Microscopic images of the LU-HNSCC-26 cell line. **a**. The cell line was grown until approximately 50% confluent and the photograph taken with a 10× objective. **b**. The cell line was grown as xenograft in nude mice. The section was stained with hematoxylin and eosin and the photograph taken with a 20× objective. **c**. The original tumor stained with hematoxylin and eosin. **d**. Xenograft stained for p16 protein. **e**. The original tumor stained for p16 protein

**Fig. 2:**
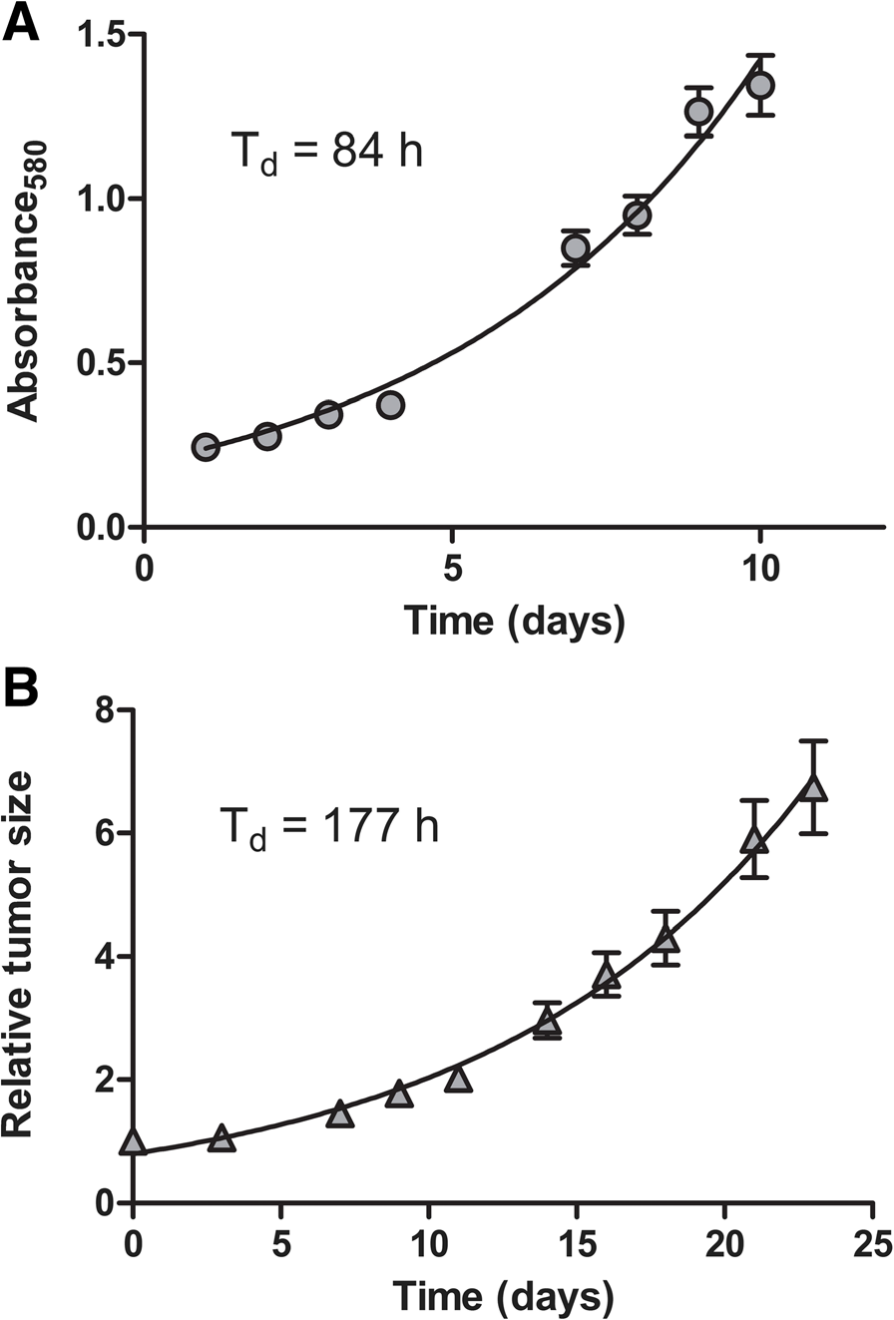
Logarithmic growth of LU-HNSCC-26. **a**. Cells in passage 14 were seeded in eight 96-well plates. The plates were analyzed by the sulphorhodamine **b** (SRB) method consecutively at the indicated time points. Error bars indicate standard error of the mean. The data points were fitted to a logarithmic equation, yielding a doubling time of 3.51 days = 84 (± 1.8) h. B. Tumors of LU-HNSCC-26 were retransplanted to 10 nude mice, two tumors per mouse. Tumors were measured with calipers three times per week and the relative tumor size calculated in relation to day 0, when all tumors were growing (except one that was excluded from analysis). Error bars indicate standard error of the mean. The data points were fitted to a logarithmic equation, yielding a doubling time (Td) of 7.38 days = 177 (± 27) h

Approximately 19 months (8 passages) after the initial seeding, the cell line was visibly free from fibroblasts and displayed a stable growth rate (Fig. 1a) and was denominated LU-HNSCC-26. The doubling time was determined to be 84 h (Fig. 2a).

For future identification and authentication of the cell line, STR profiling was performed using 16 loci (Table 2). No exact matches were found when comparing with other analyzed cell lines in the DSMZ repository [27].

**Table 2:** STR analysis

LU-HNSCC-26		
AMEL	X	Y
CSF1PO	11	13
D13S317	11	11
D16S539	13	14
D18S51	13	13
D19S433	13	14
D21S11	28	28
D2S1338	16	16
D3S1358	15	15
D5S818	10	12
D7S820	13	13
D8S1179	14	15
FGA	20	22
TH01	9	10
TPOX	8	11
vWA	19	21

**Fig. 3:**
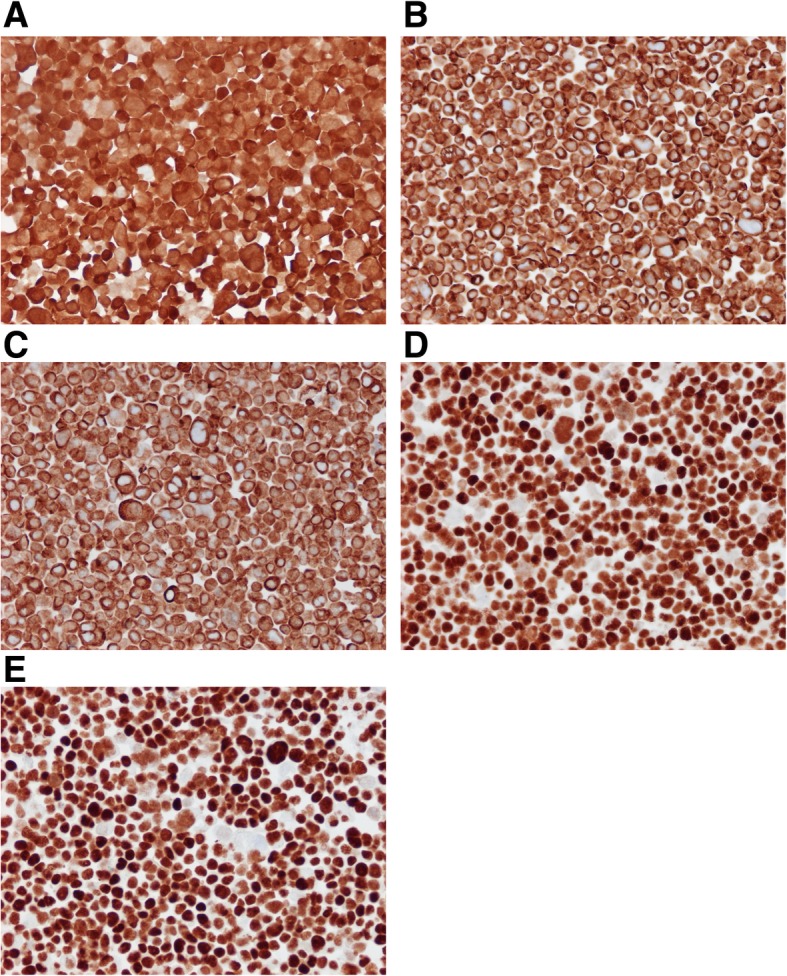
Cell pellets of LU-HNSCC-26 were sectioned in 3-μm slices and stained with antibodies against **a**. p16, **b**. CKAE1/AE3, **c**. CK5, **d**. p63 and E. p40. All photos were taken with a 20× objective

Cell pellets were sectioned and stained for p16 and epithelial markers. The cells were p16-positive (cytoplasmic and nuclear staining) (Fig. 3a). They were also positive for CKAE1/AE3 and CK5 (cytoplasmic staining) (Fig. 3b and c), and p63 and p40 (strong nuclear staining) (Fig. 3d and e), establishing the squamous epithelial origin of the cells.

The LU-HNSCC-26 cells were injected subcutaneously in nude mice resulting in xenografts that could be successfully retransplanted to new mice. The doubling time of tumor growth was determined to be 177 h (Fig. 2b). The histology of the resulting tumors was compared with that of the original tumor. Both tumors showed non-keratinizing morphology, growing in solid sheets and trabeculae. Scattered mitoses and apoptotic cells was seen in both, whereas prominent tumor necrosis was only is observed in the xenograft (Fig. 1b and c). Both xenograft and original tumor were strongly positive for p16 (Fig. 1d and e).

### Detection and characterization of HPV

**Fig. 4:**
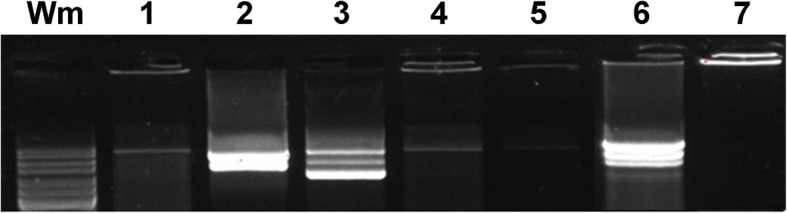
Amplicons of long range HPV16 PCR from established cell line from a tonsil carcinoma at passage 2 (lane 1) and 8 (lanes 2–5 show 2-fold dilution series of template input to PCR). Lane 1: passage 2, input 15 ng, lane 2: passage 8, 37 ng, lane 3: passage 8, 18 ng, lane 4: passage 8, 9 ng. lane 5: passage 8, 4.5 ng. Lane 6: positive control, 5000 copies of cloned HPV16. Lane 7: negative control, water. Wm: Gene Ruler 100–10,000 bp (Thermo Fisher Scientific). The arrow indicates the position of an amplicon of 7900 bp

At passage two and eight the viral DNA load was 14.8, coefficient of variation (CV%) 6.6, and 19.5 (CV% 3.7) copies per cell, respectively. Long range PCR for detection of the complete HPV16 genome from passage two and eight demonstrated amplicons of approximately 8 kb (Fig. 4). Episomal HPV16 was present in the cell line with E2/E7 ratios of 0.8 (CV% 1.7) and 1.1 (CV% 2.6) from passage two and eight, respectively. The viral expression manifested 1.0 (CV% 0.1) E7 mRNA copies per HPV16 genome of passage two.

**Fig. 5:**
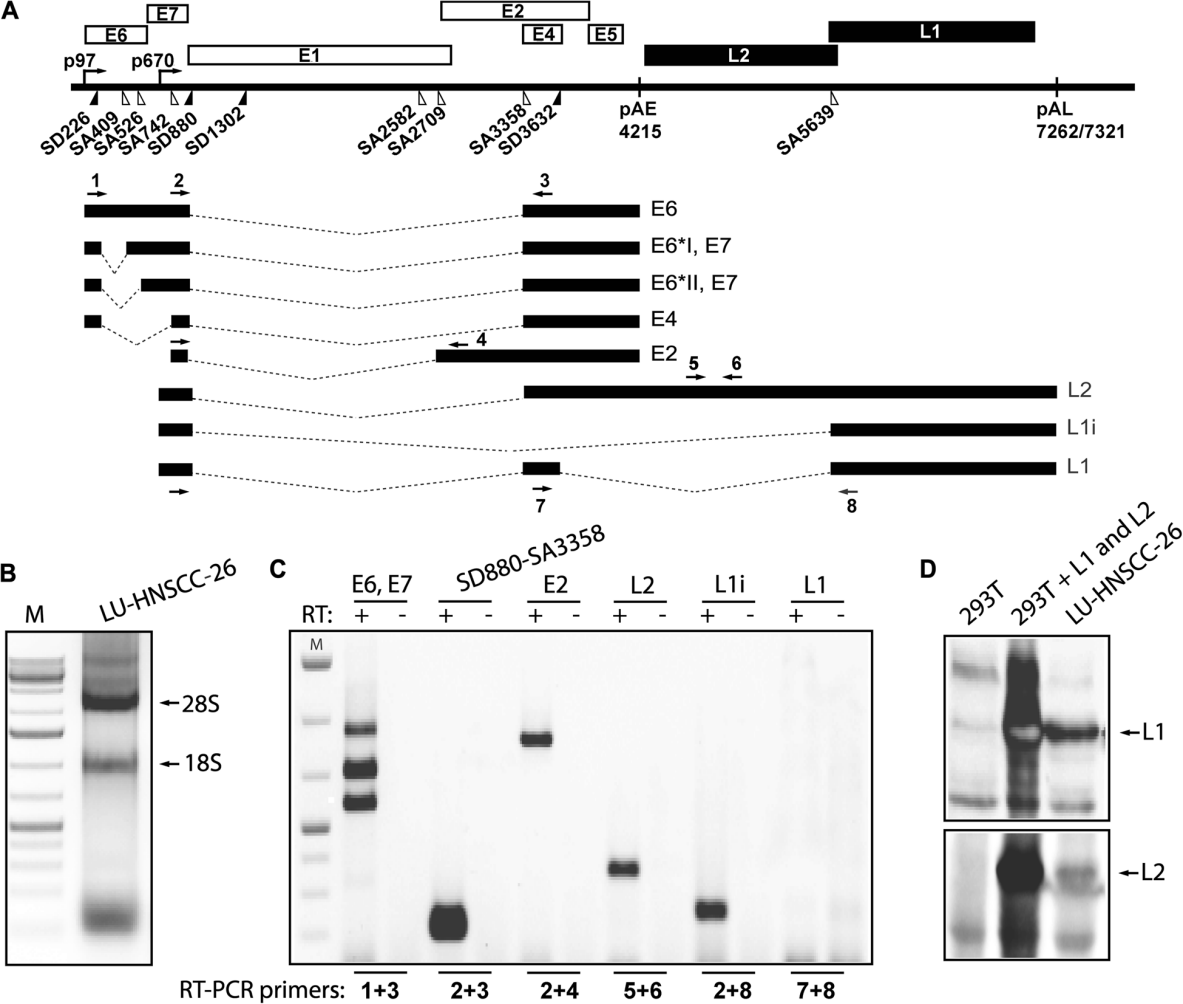
**a**. Schematic representation of the HPV16 genome. Rectangles represent open reading frames, promoters p97 and p670 are indicated as arrows, and filled and open triangles represent 5′- and 3′-splices sites respectively [39]. HPV16 early and late polyA signals pAE and pAL are indicated. The mRNAs produced by HPV16 cells are indicated below the genome and RT-PCR primers are indicated as arrows and numbered. RT-PCR primers are listed in Table 1. **b**. Total RNA extracted from LU-HNSCC-26 cells. 28S and 18S ribosomal RNAs are indicated. C. RT-PCR on total RNA extracted from the LU-HNSCC-26 cell line with the indicated primer pairs. D. Western blot with antibody against the HPV16 L1 or L2 protein on cell extracts from 293 T cells, 293 T cells transfected with a codon modified HPV16 L1 expression plasmid or LU-HNSCC-26 cells

To investigate if correctly spliced HPV16 mRNAs were produced, we extracted total RNA (Fig. 5b) and performed RT-PCR with various HPV16 specific primers (Fig. 5a). We found production of mRNAs encoding full-length E6, E6*I, E6*II and E7 proteins. The E7 mRNA spliced from SD226 to SA409 was the most abundant of the early mRNAs (Fig. 5c).). Both E2 mRNAs and mRNA spliced from SD880 to SA3358 were detected (Fig. 5c), confirming that episomal copies of the HPV16 genome were present and transcriptionally active. We detected mRNAs spliced from SD880 to the exclusively late 3′-splice site SA5639, suggesting that L1i mRNAs were produced (Fig. 5c), and L2 mRNAs were also detected. These results suggested that HPV16 late proteins L1 and L2 could be produced in the LU-HNSCC-26 cell. We therefore performed a Western blot which confirmed the presence of L1 and L2 proteins in the LU-HNSCC-26 cells (Fig. 5d), albeit at very low levels.

**Fig. 6:**
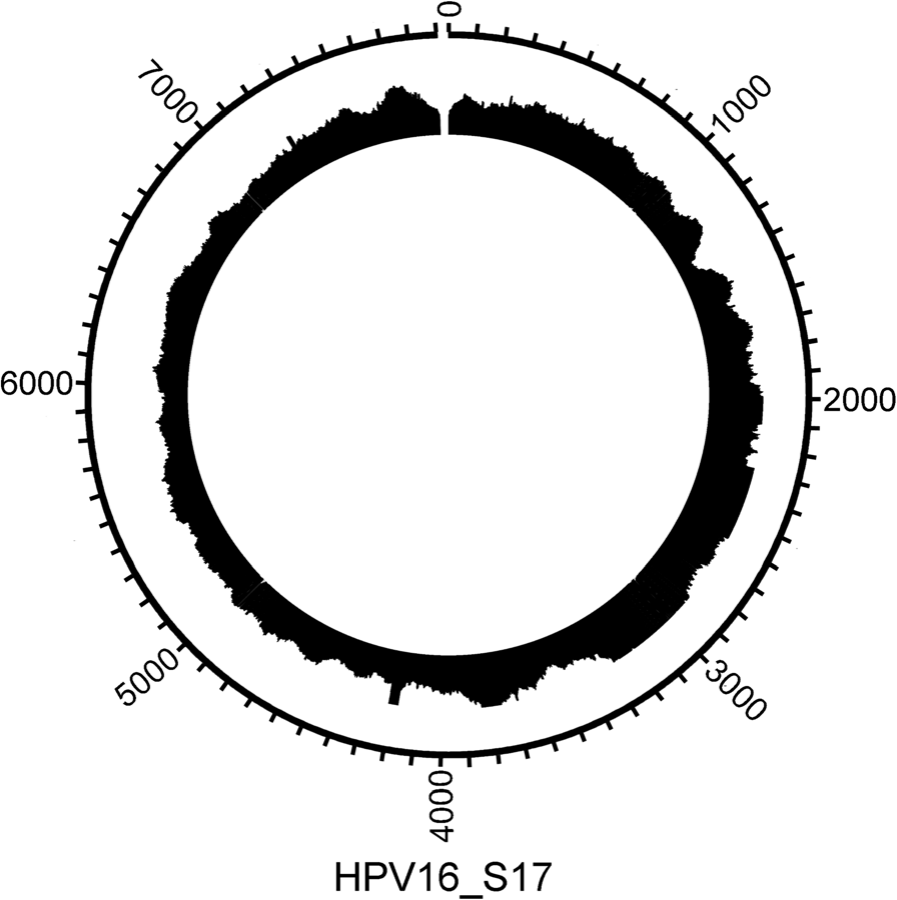
Coverage plot of the de novo HPV16 sequence of LU-HNSCC-26. Coverage was at minimum 134

From passage two, the complete DNA sequence of HPV16 was obtained by NGS with a minimum nucleotide coverage of 134 (Fig. 6). The HPV16 genome comprised 7904 bp, GenBank accession KY994539. The genomic organization showed the characteristic features shared by HPV types. The HPV16 genome showed closest identity (99%, 7900/7906) to the HPV16 strain CU4 (GenBank accession FJ610149), originally isolated from a uterine cervical cytology sample with CINI. In comparisons to this isolate, our HPV16 demonstrated a deletion of two bp in the second non-coding region between the E5 and L2 ORFs. Furthermore, within the E1 ORF at nucleotide position 1376, a guanosine was present instead of adenosine, translating to cysteine instead of tyrosine in the putative E1 protein.

### Genetic characterization of the cell line

The karyotype was determined to be 43–47,XY,del(2)(q33), del(3)(p21),der(4)t(4;5)(q35;q31), add(5) (q35),-7,-14,add (15) (p11),?i(16)(q10),-21,+mar[cp6]/44–47,idem,i(8)(q10)[cp3] (see karyogram in Additional file 1).

The gene *TP53* was wild-type but homozygous for a single nucleotide polymorphism chr17:7579472G > C, giving a proline to arginine substitution at position 72 (see partial sequencing results in Additional file 2).

### Treatment sensitivities

**Fig. 7:**
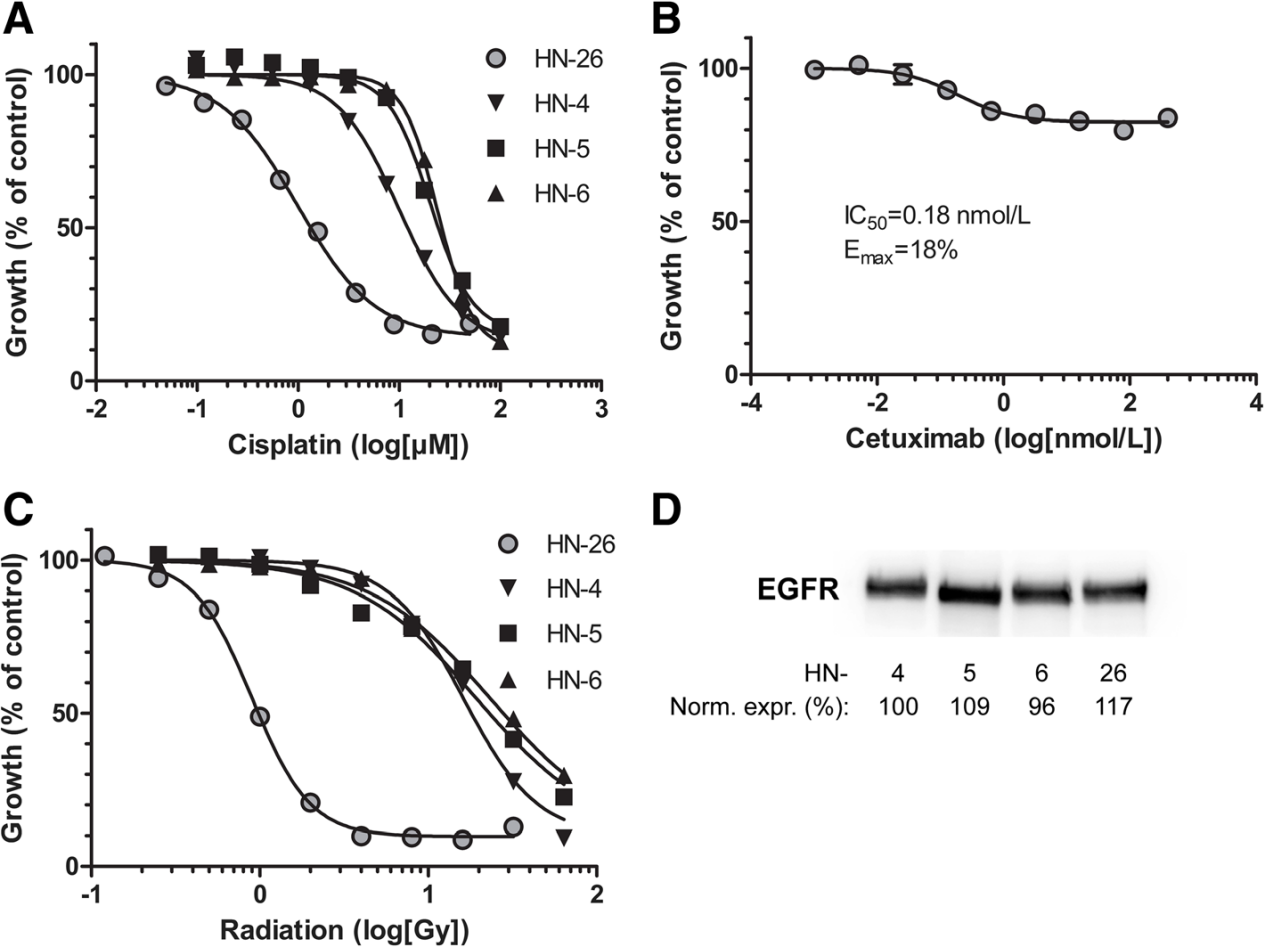
Inhibition of LU-HNSCC-26 growth using cisplatin (**a**), cetuximab (**b**) and ionizing radiation (**c**), and expression of epidermal growth factor receptor (EGFR) (**d**). A. The HPV-positive LU-HNSCC-26 (LU-HNSCC-26, grey circles) and three HPV-negative HNSCC cell lines (black symbols) were treated with increasing concentrations of cisplatin for one hour. After 5 days the cells were analyzed by the sulphorhodamine B (SRB) method. The data points were fitted to a sigmoidal dose-response equation yielding inhibitor concentration (IC_50_) values as indicated in Table 3. B. LU-HNSCC-26 cells were seeded in 96-well plates and treated with increasing concentrations of cetuximab for 5 days. Growth was analyzed with the SRB method and the data was treated as above. C. The four cell lines were irradiated with increasing doses and analyzed by the SRB method when the untreated controls were approximately 90% confluent. The data points were fitted to a sigmoidal dose-response equation yielding inhibitor dose (ID_50_) values as indicated in Table 3. In all panels (**a**-**c**), error bars indicate standard error of the mean. D. Cell lysates were prepared from all cell lines and probed with anti-EGFR. Expression was normalized to protein load and indicated as percentage of the expression in HN-4

The sensitivities to commonly used treatments were assessed in vitro and compared to three HPV-negative HNSCC cell lines. LU-HNSCC-26 was considerably more sensitive to cisplatin than the other HNSCC cell lines tested (Fig. 7a and Table 3). The IC_50_ for LU-HNSCC-26 (0.99 μmol/mL) was 10–20 times lower than those of the other cell lines.

**Table 3:** Sensitivity to cisplatin, cetuximab and radiation

	Cisplatin	Cetuximab	Radiation
Cell line	IC_50_ (μmol/L)^a^	E_max_ (%)^a^	ID_50_ (Gy)^a^
LU-HNSCC-26	0.99 (0.89–1.1)	18 (17–19)	0.90 (0.85–0.95)
LU-HNSCC-4	10 (9.0–11)	*29 (27–32)* ^*b*^	16 (15–17)
LU-HNSCC-5	20 (19–22)	*74 (68–80)* ^*b*^	19 (18–21)
LU-HNSCC-6	24 (23–26)	*51 (43–60)* ^*b*^	23 (22–25)

^a^(95% confidence interval)

^b^From reference [28]

For cetuximab, we compared the maximum inhibition (E_max_) of the drug instead of the IC_50_. LU-HNSCC-26 was only inhibited to a maximum of 18% (Fig. 7b and Table 3), which was less than the other cell lines [28]. This difference could not be correlated with the EGFR expression as assessed by Western blotting showing a similar expression in all four cell lines (Fig. 7d).

As for cisplatin, LU-HNSCC-26 was considerably more sensitive to radiation compared to the other cell lines (Fig. 7c and Table 3). The ID_50_ for LU-HNSCC-26 (0.90 Gy) was 18–26 times lower than those of the other cell lines.

## Discussion

In the current work we demonstrated the in vitro establishment of an HPV16-positive cell line, LU-HNSCC-26, derived from a tonsil carcinoma. The cell line manifested episomal HPV16 with active expression of viral mRNA. The *TP53* gene was wild type and the cell line displayed a relatively simple karyotype including loss of 3p, 4q, and 7p as well as isochromosomes, which are common findings in HPV-positive HNSCC-cases, that often have less complex karyotypes with fewer signs of chromosomal instability compared to HPV-negative ones [29]. The doubling time in vitro was 84 h which is approximately 3 times slower than most other HNSCC cell lines established in our laboratory. The cell line was also grown as a xenograft in nude mice and the tumors displayed a histology that closely matched that of the original tumor. The cell line showed high sensitivity to cisplatin and to radiation but was less sensitive to treatment with cetuximab, compared with that of three HPV-negative HNSCC cell lines previously described by us [17].

It is widely accepted that HPV infection plays an etiological role in many oropharyngeal cancers and there is ample evidence that HPV-positivity is coupled to increased survival and better prognosis. As a result of this, there are currently several clinical trials addressing the question whether it is possible to de-intensify the treatment for this patient group to reduce the toxicities while preserving the efficacy [30]. However, as the carcinogenic process most certainly is different for HPV-driven tumors, there is also an opportunity to increase the efficacy of treatment for these patients by targeting mechanisms specific to HPV-positive cancers.

Well characterized cellular and animal models are valuable tools for efficient treatment development. Such models can provide fast and efficient testing of novel drugs and combinations of drugs or other treatment modalities. So far, no in vitro cell line has been established from HPV-positive tonsil cancer and only few from other head and neck locations [7, 8]. Importantly, there are clear indications that, in contrast to OSCC, the outcome for HPV-positive cases in sites other than oropharynx is not favorable compared to HPV-negative ones [9–11]. Cell lines derived from other sites, though probably mirroring the properties of the originating tumors [31], might therefore not correctly represent the properties of HPV-positive OSCC. Thus, the presented cell line provides an important step forward in the quest for improved therapies against HPV-positive OSCC and seems to be representative for this cancer type in the measured variables.

The presence of the episomal state of the HPV16 in the LU-HNSCC-26 cell line, as demonstrated by the complete circular genome of HPV 16 and as well as the intact HPV16 E2 mRNA, is similar to that of our previously established HPV16-positive tonsil carcinoma xenograft, which manifested episomal HPV16 and integrated HPV16 simultaneously [18]. Overall, the episomal form of HPV16, either as pure episomal or as a mixed status of both integrated and episomal HPV16, is in predominance in tonsil carcinomas [13, 14, 32]. The relatively high proportion of intact episomal HPV16 in these lesions may lead to repression of the viral oncogene transcription via expression of intact HPV16 E2-protein, which binds to conserved sites of the upper regulatory region of HPV16 genome [33]. Interestingly, the latency period between HPV infection and oropharyngeal cancer development has been estimated to be up to 30 years whereas the corresponding period for cancer of the cervix uteri is around 20 years [34]. Speculatively, the longer latency period for HPV-positive tonsil carcinomas compared to that of cervical cancer might partly be explained by a predominance of the episomal HPV16 among tonsil carcinomas.

The HPV16 positive LU-HNSCC-26 cell line manifested wild-type *TP53*, homozygous for arginine at codon 72 of the *TP53*. Interestingly, this arginine form of the gene product p53 has been shown to be more susceptible to HPV16 E6 mediated degradation than the proline form [35]. In addition, the arginine form of p53 was found to be associated with HNSCC, but not with worse prognosis of the disease [36]. Furthermore, Habbous et al. reported in a meta-analysis that the arginine variant of p53 was associated with progression of squamous intraepithelial lesions to cervical cancer only in the presence of HPV positivity [37]. Further studies are required to investigate the link between the arginine variant of p53 and susceptibility for development of tonsil carcinoma among HPV-infected individuals.

The p53 protein is inactivated by the HPV16 E6 protein [38]. Here we demonstrated HPV16 mRNAs encoding full-length E6 protein as well as E6*I, E6*II and E7, strongly supporting the idea that p53 is inactivated by HPV16 E6 protein in LU-HNSCC-26 cells. The splicing pattern of the various E6 and E7 mRNAs is reminiscent of the splicing pattern observed in HPV16-positive cervical cancer cell lines with relatively low levels of the mRNA encoding the full-length E6 protein, while the most abundant early mRNA is coding for E6*I and E7. The relative levels of the various HPV16 E6 and E7 mRNAs are determined by cis-acting RNA elements on the HPV16 mRNAs, and by cellular proteins that bind to these elements to control splicing efficiency [39, 40]. One may speculate that the levels of these cellular splicing regulatory proteins are similar in cervical cancer and tonsil cancer cells. Interestingly we detected HPV16 L1 and L2 proteins from the LU-HNSCC-26 cell line. However, initial attempts to identify virus particles from the cell line have been unsuccessful (data not shown, personal communication H. Faust).

In this work we attempted to set up cell lines from 27 tonsil tumors of which 23 were HPV positive but only one of these actually resulted in an established cell line. This success rate is considerably lower than that for HNSCC in general, previously reported to be around 33% in our lab [17]. In our hands, the establishment of HPV-positive tonsil cancer cell lines has thus been much more difficult than that of other HNSCC tumor types. The low number of HPV-negative tumors included in this study precludes any conclusion about the take rate of these cells.

There are around 14 tonsil cell lines described in the literature [41] of which, as far as we know, all are negative for HPV, though one in vitro cell line from tonsil fossa with only integrated HPV16 was recently reported [42]. Overall, this implies that HPV-positive tonsil cancers are indeed less prone to grow in vitro than the HPV-negative ones, which is surprising considering the ease with which HPV-positive cell lines have been established from cervical cancers.

Conflicting results regarding the sensitivity of HPV-positive HNSCC cell lines to cisplatin have previously been reported indicating either similar sensitivities between HPV-positive and negative cell lines [43] or higher sensitivities in HPV-positive cell lines [44]. This is remarkable as several of the cell lines used were identical between the two studies, but indicates that the different results depend on methodological issues. In our work, LU-HNSCC-26 was clearly more sensitive to cisplatin than the three HPV-negative HNSCC cell lines used for comparison, with the IC_50_ being 10 to 20 times lower (Table 3). In combination with the low sensitivity of the cell line to cetuximab (maximum 18% inhibition), this is interesting considering the current discussion on deescalating therapies by omitting cisplatin or exchanging with targeting drugs such as cetuximab; an alternative not suggested by the present results.

## Conclusions

To the best of our knowledge, we have established the first in vitro tonsil carcinoma cell line containing episomal HPV16. The cell line demonstrated high sensitivity to radiation and cisplatin treatment. The cell line provides an important experimental system of episomal HPV16-positive tonsil carcinomas in an era of increasing incidence of tonsil cancer in several countries.

## Additional files

Additional file 1: Karyogram from LU-HNSCC-26. (PDF 73 kb)

Additional file 2: Partial sequencing results for *TP53*. The LU-HNSSC-26 is homozygous for the single nucleotide polymorphism chr17:7579472G > C (rs1042522). Partial sequencing results for *TP53* mapped against GRCh37 (top) and Sanger sequencing electropherogram (bottom) with the rs1042522 indicated by arrows. (PDF 172 kb)

## Abbreviations

CV%: coefficient of variation
DMEM: Dulbecco’s modified Eagle’s medium
EGFR: epidermal growth factor receptor
E_max_: maximum inhibition
FBS: fetal bovine serum
HNSCC: head and neck squamous cell carcinoma
HPV: human papillomavirus
IC_50_: half maximal inhibitory concentration
ID_50_: half maximal inhibitory dose
MGP-PCR: modified general primer polymerase chain reaction
NGS: next-generation sequencing
ORF: open reading frame
OSCC: oropharyngeal squamous cell carcinoma
PBS: phosphate buffered saline
PCR: polymerase chain reaction
RIPA: radioimmunoprecipitation assay
SRB: sulphorhodamine B
STR: single tandem repeat
T_d_: doubling time
WGA: whole genome amplified
