# Supplementary figures and images for: A novel human in vitro papillomavirus type 16 positive tonsil cancer cell line with high sensitivity to radiation and cisplatin
Source: BMC Cancer. 2019 Mar 25;19:265. doi: 10.1186/s12885-019-5469-8 (PMC6434888; doi:10.1186/s12885-019-5469-8)

## LU-HNSCC-26

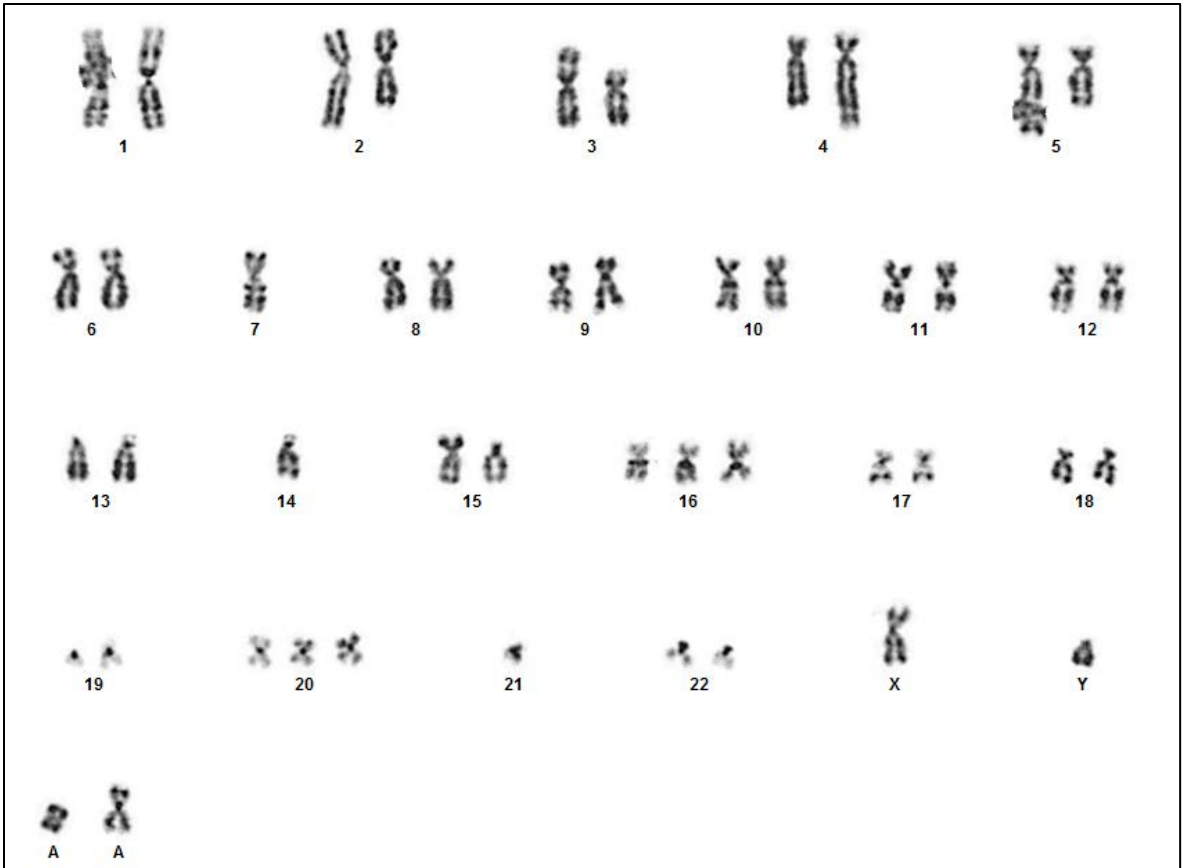

47,XY,del(2)(q33),del(3)(p21),der(4)t(4;5)(q35;q31),add(5)(q35),-7,-14,add(15p11),?i(16)(q10),-21,+mar[cp6]/44-47, idem, i(8)(q10)[cp3]

Supplement: Supplementary file 1 — Karyogram from LU-HNSCC-26. (PDF 73 kb) [file 12885_2019_5469_MOESM1_ESM.pdf]
