# Supplementary material for: A novel human in vitro papillomavirus type 16 positive tonsil cancer cell line with high sensitivity to radiation and cisplatin
Source: BMC Cancer. 2019 Mar 25;19:265. doi: 10.1186/s12885-019-5469-8 (PMC6434888; doi:10.1186/s12885-019-5469-8)

chr17:7579472G>C (rs1042522)

```
0000201 tggtaggttttctgggaagggacagaagatgacaggggccaggagggggc 0000250
>>>>>>> |||||||||||||||||||||||||||||||||||||||||||||||||| >>>>>>>
7579376 tggtaggttttctgggaagggacagaagatgacaggggccaggagggggc 7579425

0000251 tggtgcaggggccgccggtgtaggagctgctggtgcaggggccacgcggg 0000300
>>>>>>> ||||||||||||||||||||||||||||||||||||||||||||| |||| >>>>>>>
7579426 tggtgcaggggccgccggtgtaggagctgctggtgcaggggccacggggg 7579475

0000301 gagcagcctctggcattctgggagcttcatctggacctgggtcttcagtg 0000350
>>>>>>> |||||||||||||||||||||||||||||||||||||||||||||||||| >>>>>>>
7579476 gagcagcctctggcattctgggagcttcatctggacctgggtcttcagtg 7579525
```

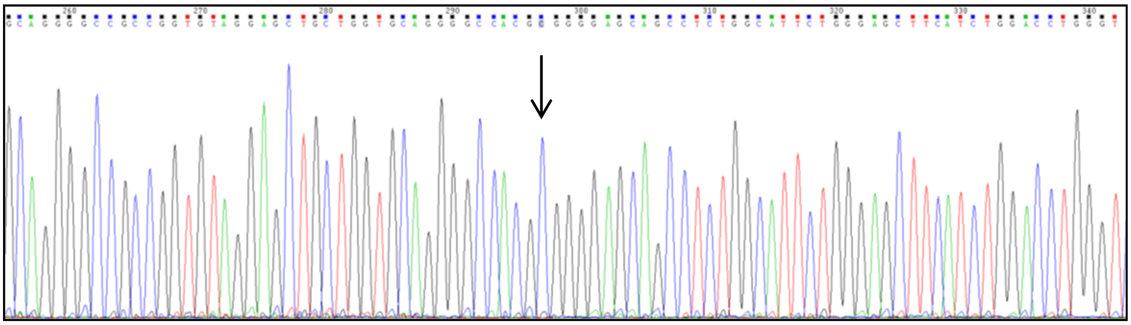

Supplement: Supplementary file 2 — Partial sequencing results for TP53. The LU-HNSSC-26 is homozygous for the single nucleotide polymorphism chr17:7579472G > C (rs1042522). Partial sequencing results for TP53 mapped against GRCh37 (top) and Sanger sequencing electropherogram (bottom) with the rs1042522 indicated by arrows. (PDF 172 kb) [file 12885_2019_5469_MOESM2_ESM.pdf]
